# Addition to the study of the genus *Dusona* (Hymenoptera, Ichneumonidae, Campopleginae) in Korea with description of a new species and key to the Korean species

**DOI:** 10.3897/zookeys.424.7546

**Published:** 2014-07-08

**Authors:** Jin-Kyung Choi, Jong-Wook Lee

**Affiliations:** 1 Department of Life Sciences, Yeungnam University, Gyeongsan, 712-749, South Korea

**Keywords:** *Dusona koreana* sp. n., taxonomy

## Abstract

Korean species of the genus *Dusona* Cameron (Hymenoptera: Ichneumonidae: Campopleginae) are reviewed. Twenty seven species of *Dusona* are reported from South Korea, including 12 previously unrecorded species, *D. bellipes* (Holmgren, 1872), *D. bicoloripes* (Ashmead, 1906), *D. chabarowski* Hinz & Horstmann, 2004, *D. cultrator* (Gravenhorst, 1829), *D. japonica* (Cameron, 1906), *D. mactatoides* Hinz, 1994, *D. scalprata* Horstmann, 2004, *D. sasayamae* Hinz & Horstmann, 2004, *D. oblitera* (Holmgren, 1872), *D. obtutor* Hinz, 1994, *D. auriculator* Aubert, 1964, *D. longicauda* (Uchida, 1928), and a new species, *D. koreana* **sp. n.** An illustrated key to Korean species of *Dusona* provided.

## Introduction

The subfamily Campopleginae includes more than 2,000 valid species worldwide. Yu et al. (2012) listed 33 species of 11 genera in Korea, 431 species in the Eastern Palaearctic region, and 2,102 species of 66 genera worldwide. Among them, *Dusona* is the largest genus of Campopleginae, cosmopolitan with 440 described species (Yu et al. 2012). Taxonomic study of Korean Campopleginae was initiated by Matsumura (1926). Since the first record of Korean campoplegine species by Matsumura, there have been only a few reports on Campopleginae by Kim (1955). Since then intensive study of Korean Campopleginae has only been performed in our recent study. Some species of *Dusona* have been reported by Hinz and Horstmann (2004) and Choi and Lee (2008).

In this study a new species, *Dusona koreana* sp. n., is described. We also report 12 species new for the Korean fauna: *Dusona bellipes* (Holmgren, 1872), *Dusona bicoloripes* (Ashmead, 1906), *Dusona chabarowski* Hinz & Horstmann, 2004, *Dusona cultrator* (Gravenhorst, 1829), *Dusona japonica* (Cameron, 1906), *Dusona mactatoides* Hinz, 1994, *Dusona scalprata* Horstmann, 2004, *Dusona sasayamae* Hinz & Horstmann, 2004, *Dusona obliterata* (Holmgren, 1872), *Dusona obtutor* Hinz, 1994, *Dusona auriculator* Aubert, 1964 and *Dusona longicauda* (Uchida, 1928).

We also provide a description with photographs of the new species, comparative illustrations of all Korean species of *Dusona*, including habitus photographs, and an identification key to all Korean species.

## Materials and methods

Specimens used in this study were collected by sweeping and Malaise trapping, and are deposited in the animal systematic laboratory of Yeungnam University (YNU, Gyeongsan, Korea). Specimens were photographed using an AxioCam MRc5 camera attached to a stereo microscope (Zeiss SteREO Discovery. V20; Carl Zeiss, Göttingen, Germany), processed using AxioVision SE64 software (Carl Zeiss), and optimized with a Delta imaging system (i-solution, IMT i-Solution Inc. Vancouver, Canada). Some specimens examined in this study were loaned by the ZSM (Zoologisches Staatsammlung, München, Germany). The morphological terminology is mostly that of Gupta and Maheshwary (1977). Distribution data and host records are taken from Yu et al. (2012) and Horstmann (2011).

Abbreviations are as follows. TD, type depository; TS, type species; CNC, Canadian National Collections, Centre for Land and Biological Resources Research, Agriculture Canada, Ottawa, Ontario, K1A 0C6, Canada; DEI, Deutsches Entomologisches Institut, Schicklerstrasse 5, D-16225; GUPTA, Entomology & Nematology Department, University of Florida, Gainesville, Florida, 32611, U.S.A.; HU, Hokkaido University, Faculty of Agriculture, Entomological Institute, Sapporo, Japan; MCZ, Museum of Comparative Zoology, Harvard University, Cambridge, Massachusetts, 02138, U.S.A.; MLSU, Zoological Museum, Moscow Lomonosov State University, Moscow, Russia; MRSN, Museo Regionale di Scienze Naturali, Via Giolitti 36, I-10123 Torino, Italy; MZ, Musée Zoologique, Place Riponne, CH-1000 Lausanne, Switzerland; NHM, The Natural History Museum, Department of Entomology, Cromwell Road, London, England, SW7 5BD, United Kingdom; NM, Naturwissenschaftliche Sammlungen der Stadt Krefeld, Brempter Hof, D-47829 Krefeld-Uerdingen, Germany; NR, Naturhistoriska Riksmuseet, Sektionen för Entomologi, S-104 05 Stockholm, Sweden; SAWON, Department of Forest Protection and Ecology, Warsaw Agricultural University, ul. Rakowiecka 26/30, 02-528 Warszawa, Poland; TMA, Termeszettudomanyi Muzeum Allattara, Barossa-Utea 13, Budapest H-1088, Hungary; USNM, National Museum of Natural History, Smithsonian Institute, Washington, D.C., 20560, U.S.A.; UZM, Universitets Zoologiske Museum, Universitetsparken 15, Copenhagen, Denmark; YU, Yale University, Peabody Museum, New Haven, Connecticut, 06511, U.S.A.; ZI, Zoological Institute, Academy of Sciences, St. Petersburg 199034, Russia; YNU, Animal systematic laboratory of Yeungnam University, Gyeongsan, Korea; ZSM, Zoologisches Staatsammlung, D 81247, München, Germany; GW, Gangwon-do; GG, Gyeonggi-do; GB, Gyeongsangbuk-do; GN, Gyeongsangnam-do; JB, Jeollabuk-do; JN, Jeollanam-do; JJ, Jeju-do.

## Results

### Family Ichneumonidae Latreille, 1802
Subfamily Campopleginae Förster, 1869

#### Genus Dusona

Taxon classificationAnimaliaHymenopteraIchneumonidae

Cameron, 1901

Dusona Cameron, 1901: 107. TS: *Dusona stramineipes* Cameron
Delopia Cameron, 1903: 304. TS: *Delopia cariniscutis* Cameron = *Dusona cariniscutis* (Cameron, 1903)
Campoplegidea Viereck, 1912: 633. TS: *Campoplex oxyacanthae* (Boie, 1855) = *Dusona mercator* (Fabricius, 1793)
Pseudocasinaria Viereck, 1912: 644. TS: *Casinaria americana* Ashmead = *Dusona americana* (Ashmead, 1890) = *Dusona annexa* (Förster, 1868)
Thymarimorpha Viereck, 1913: 384. TS: *Thymarimorpha platygastra* Viereck = *Dusona gnara* (Cresson, 1874)
Viereckiana Strand, 1914: 163-164.
Zachrestinus Enderlein, 1921: 38. TS: *Zachrestinus fractocristatus* Enderlein = *Dusona fractocristata* (Enderlein, 1921)
Idiosomidea Viereck, 1925: 271. TS: *Campoplex photomorphus* (Viereck, 1905) = *Dusona bellula* (Dalla Torre, 1901)
Neodelopia Benoit, 1957: 314. TS: *Neodelopia pauliani* Benoit = *Dusona pauliani* (Benoit, 1957)
Kartika Gupta & Gupta, 1976: 460. TS: *Kartika aspera* Gupta & Gupta = *Dusona aspera* (Gupta & Gupta, 1976)

##### Diagnosis.

Inner margin of eye with emargination opposite antenna socket; clypeus weakly convex, truncate or blunt; areola and petiolar areas of propodeum not separated by carina; propodeum with elongate spiracle; fore wing with large, usually rhombic areolet, pointed or stalked; discoidella reaching nervellus or detached; glymma of petiole present, vestigial or absent; epipleurum of 3^rd^ tergum not separated by crease or sometimes partly separated; metasomal segments usually reddish brown and partly black or sometimes mostly black.

##### Distribution.

Worldwide.

##### Key to the species of genus *Dusona* from Korea

**Table:** 

1	Epipleurum separated from the 3^rd^ tergum, the crease with black line (Fig. 7D)	2
–	Epipleurum not separated from the 3^rd^ tergum, with lateral black line above the anterior ventrolateral edge or without lateral black line (Figs 7J, 7K)	13
2	Ovipositor upcurved and longer than hind tibia (Fig. 3L)	*Dusona longicauda*
–	Ovipositor straight and shorter than hind tibia (Fig. 1A)	3
3	Antennal flagellum with less than 40 segments. 2^nd^ recurrent vein of fore wing distad of the middle of areolet (Fig. 5D)	*Dusona maruyamator*
–	Antennal flagellum with more than 40 segments. 2^nd^ recurrent vein of fore wing basad or opposite the middle of areolet (Figs 5B, 5C)	4
4	Areolet of fore wing pentagonal shape, without stalk (Fig. 5M)	*Dusona bellipes*
–	Areolet of fore wing quadrate, with or without stalk (Fig. 5R)	5
5	Antennal carina very highly raised, with wrinkles (Fig. 8A). Central part of face with weak protuberance (Fig. 4R)	*Dusona mactatoides*
–	Antennal carina not raised or low and narrow (Fig. 8B). Face convex generally (Fig. 4F)	6
6	Mandible brown to dark brown except basal part black (Fig. 4O)	7
–	Mandible completely or partly yellow (Fig. 4K)	8
7	Mandible dark brown (Fig. 4O). Tegula black. Nervellus vertical	*Dusona chabarowski*
–	Mandible brown (Fig. 4F). Tegula yellow. Nervellus inclivous	*Dusona rugosa*
8	Petiole in front of glymma smooth (Fig. 1F)	9
–	Petiole in front of glymma at least with fine sculpture or striate (Fig. 8C)	11
9	Antennal flagellum with less than 55 segments. Clypeus with rounded apical margin	*Dusona stragifex*
–	Antennal flagellum with more than 55 segments. Clypeus with truncate apical margin	10
10	Antennal carina distinctly raised, its rim bent upwards (Fig. 8A). Nervellus vertical, intercepted in lower 0.4. 4^th^ tergum at least reddish brown anteriorly (Fig. 2B)	*Dusona celator*
–	Antennal carina low and narrow (Fig. 1C). Nervellus reclivous, intercepted in lower 0.25. Metasoma completely black posteriorly from 4^th^ tergum black completely (Fig. 1A)	*Dusona koreana* Choi & Lee, sp. n.
11	Body length longer than 17 mm. Hind femur reddish brown, sometimes marked brown or black basally (Fig. 3D). Petiole with striate in front of glymma	*Dusona cultrator*
–	Body length shorter than 16 mm. Hind femur black (Fig. 3B). Petiole with fine sculpture in front of glymma (Fig. 8C)	12
12	Clypeus with truncate apical edge (Fig. 4N). 5^th^ tergum reddish brown completely (Fig. 3B)	*Dusona bicoloripes*
–	Clypeus with concave apical edge (Fig. 4T). 5^th^ tergum reddish brown narrowly marked with black dorsally (Fig. 3H)	*Dusona sasayamae*
13	Epipleurum not separated from the 3^rd^ tergum, without anterior ventrolateral black stripe (Fig. 7K)	14
–	Epipleurum not separated from the 3^rd^ tergum, however with distinct or weak black stripe anterior ventrolateral edge (Fig. 7J)	19
14	Antennal carina raised and the rim bent upward or widened (Fig. 8A)	15
–	Antennal carina low and narrow (Fig. 8B)	17
15	Clypeus with convex apical edge. Petiole without glymma (Fig. 8D). Nervellus reclivous and not intercepted	*Dusona auriculator*
–	Clypeus with truncate or weak concave apical edge. Petiole with distinct or large deep glymma (Fig. 8C). Nervellus inclivous and intercepted	16
16	Antennal flagellum with more than 65 segments. Frons with a median longitudinal carina. Mandible dark brown	*Dusona matsumurae*
–	Antennal flagellum with fewer than 65 segments. Frons without a median longitudinal carina. Mandible yellow	*Dusona crassiventris*
17	Clypeus with convex apical edge. Areolet of fore wing small, 2^nd^ recurrent vein distad of its middle. Petiole without glymma (Fig. 5I). Body length shorter than 10 mm	*Dusona schikotani*
–	Clypeus with truncate apical edge. Areolet of fore wing large, 2^nd^ recurrent vein basad of its middle. Petiole with glymma (Fig. 5G). Body length longer than 15 mm	18
18	Antennal flagellum with more than 65 segments. Frons without median longitudinal carina	*Dusona falcator*
–	Antennal flagellum with fewer than 60 segments. Frons with strong or weak a median longitudinal carina	*Dusona obliterata*
19	Petiole with distinct or weak glymma (Fig. 8C)	20
–	Petiole without glymma (Fig. 8D	21
20	Antennal carina low, the rim weakly bent upward. Frons with incomplete median longitudinal carina. Mandible yellow. Nervellus inclivous	*Dusona glauca*
–	Antennal carina weakly raised, the rim bent upward. Frons with very high raised median longitudinal carina. Mandible brown to black. Nervellus reclivous	*Dusona ucrainica*
21	3^rd^ tergum and 4^th^ tergum black dorsally and widely reddish brown laterally	22
–	3^rd^ tergum reddish brown, 4^th^ tergum reddish brown or black with reddish brown anteriorly	23
22	2^nd^ recurrent vein of fore wing connected to middle of areolet (Fig. 5J). Nervellus vertical. Tegula yellow	*Dusona signator*
–	2^nd^ recurrent vein of fore wing basad middle of areolet (Fig. 5S). Nervellus inclivous. Tegula blackish brown	*Dusona scalprata*
23	Antennal carina weakly raised, the rim weak bent upward. Hind femur reddish brown (Fig. 2A). Body length shorter than 10 mm	*Dusona annexa*
–	Antennal carina low and narrow or very strongly raised. Hind femur black. Body length longer than 10 mm (except body length of *Dusona petiolator* 8–10 mm)	24
24	Antennal flagellum with more than 56 segments. Antennal carina strongly raised. Clypeus with convex apical edge	*Dusona okadai*
–	Antennal flagellum with less than 52 segments. Antennal carina low and narrow. Clypeus with truncate apical edge	25
25	Frons with a median longitudinal carina. Nervellus reclivous. Petiole with longitudinal striae in front of glymma	*Dusona obtutor*
–	Frons without median longitudinal carina. Nervellus inclivous. Petiole smooth in front of glymma or with weak sculpture	26
26	Mandible black. Areolet large with short stalk. 2^nd^ recurrent vein of fore wing basad middle of areolet (Fig. 5Q). 4^th^ tergum reddish brown (Fig. 3E)	*Dusona japonica*
–	Mandible yellow. Areolet large without stalk. 2^nd^ recurrent vein of fore wing distad middle of areolet. 4^th^ tergum black or reddish brown anteriorly (Fig. 2E)	*Dusona petiolator*

**Figure 1.**
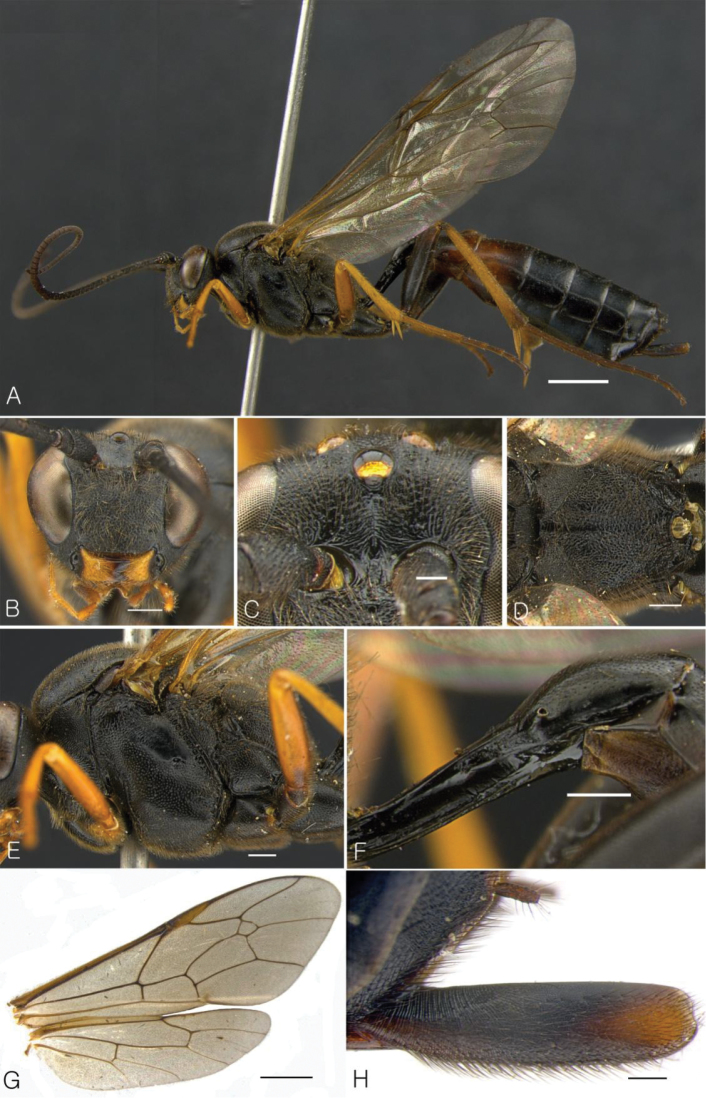
*Dusona koreana* Choi & Lee, sp. n. (female). **A** habitus in lateral view **B** head in frontal view **C** Frons **D** propodeum **E** mesosoma in lateral view **F** petiole in lateral view **G** wings **H** Ovipositor sheath. (Scale bar 2.0 mm for **A, G**; 0.5 mm for **B, D–F**; 0.2 mm for **C, H**).

**Figure 2.**
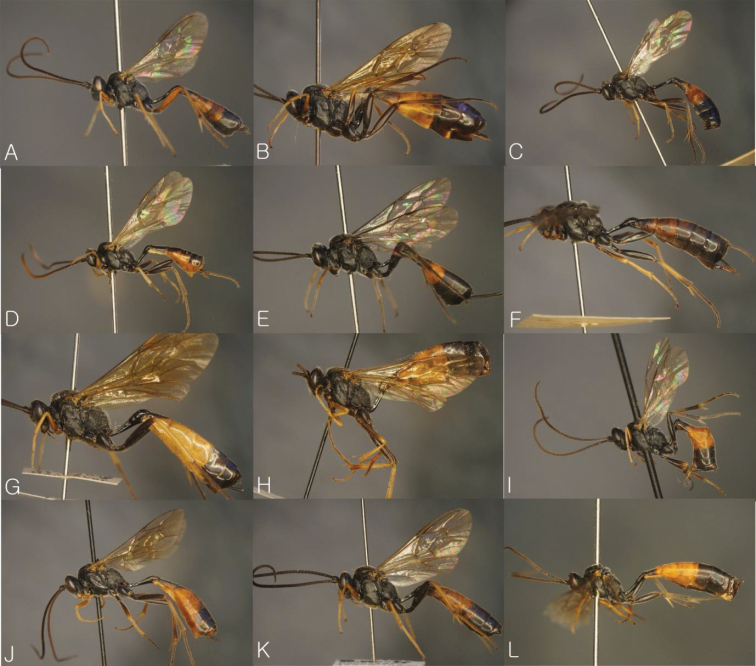
General habitus in lateral view. **A** *Dusona annexa* (=*Dusona americana*) **B** *Dusona celator* **C** *Dusona glauca* **D** *Dusona maruyamator* **E** *Dusona petiolator* **F** *Dusona rugosa* **G** *Dusona falcator* **H** *Dusona matsumurae* **I** *Dusona schikotani* **J** *Dusona signator* **K** *Dusona stragifex* **L** *Dusona ucrainica*.

**Figure 3.**
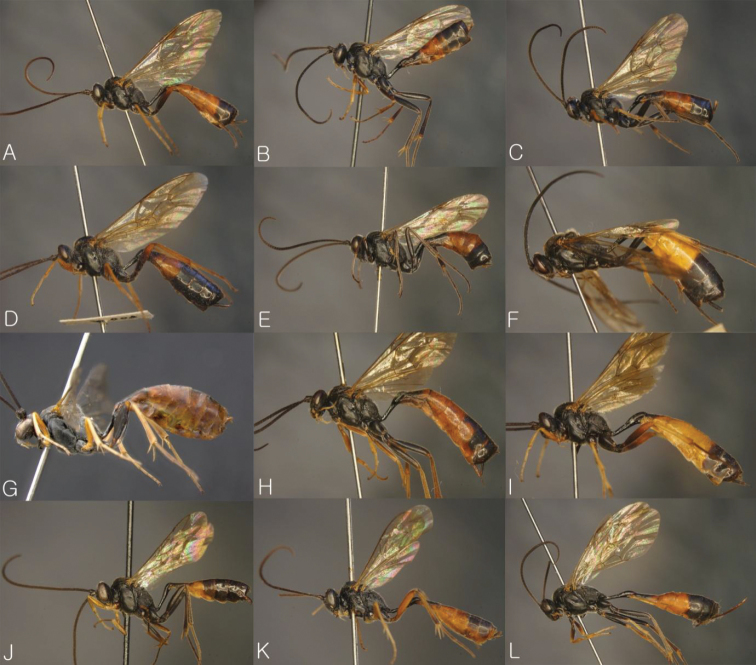
General habitus in lateral view. **A** *Dusona bellipes* **B** *Dusona bicoloripes* **C** *Dusona chabarowski* **D** *Dusona cultrator* **E** *Dusona japonica* **F** *Dusona mactatoides* **G** *Dusona scalprata* **H** *Dusona sasayamae* **I** *Dusona obliterata* **J** *Dusona obtutor* **K** *Dusona auriculator* **L** *Dusona longicauda*.

**Figure 4.**
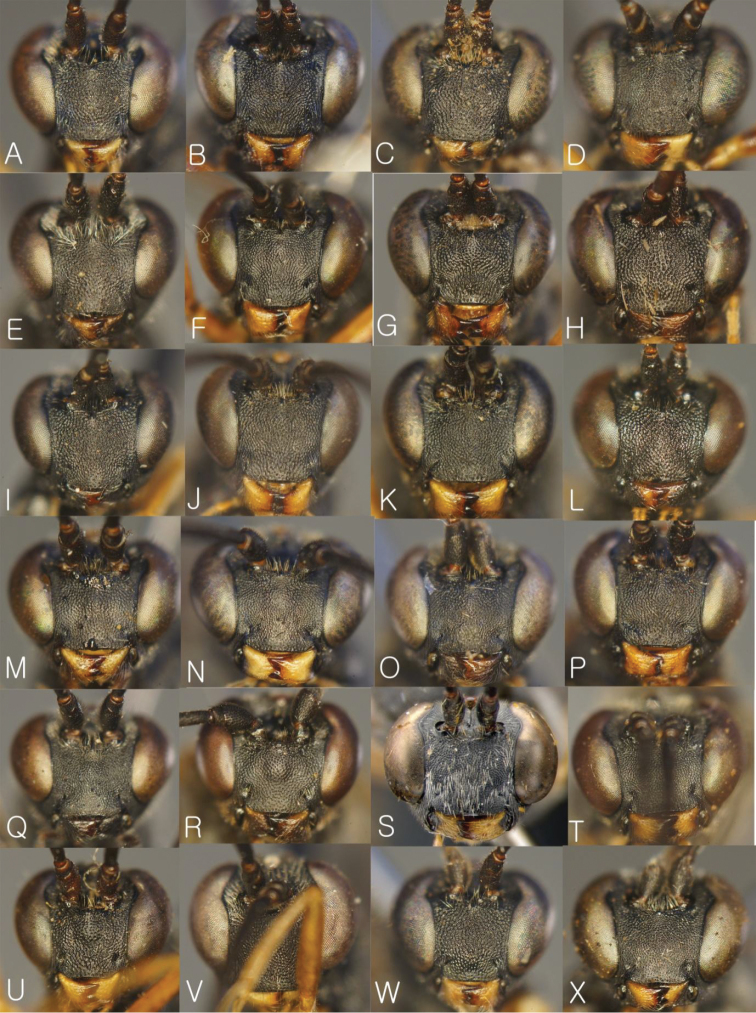
Head in frontal view. **A** *Dusona annexa* (=*Dusona americana*) **B** *Dusona celator* **C** *Dusona glauca* **D** *Dusona maruyamator* **E** *Dusona petiolator* **F** *Dusona rugosa* **G** *Dusona falcator* **H** *Dusona matsumurae* **I** *Dusona schikotani* **J** *Dusona signator* **K** *Dusona stragifex* **L** *Dusona ucrainica* **M** *Dusona bellipes* **N** *Dusona bicoloripes* **O** *Dusona chabarowski* **P** *Dusona cultrator* **Q** *Dusona japonica* **R** *Dusona mactatoides* **S** *Dusona scalprata* **T** *Dusona sasayamae* **U** *Dusona obliterata* **V** *Dusona obtutor* **W** *Dusona auriculator* **X** *Dusona longicauda*.

**Figure 5.**
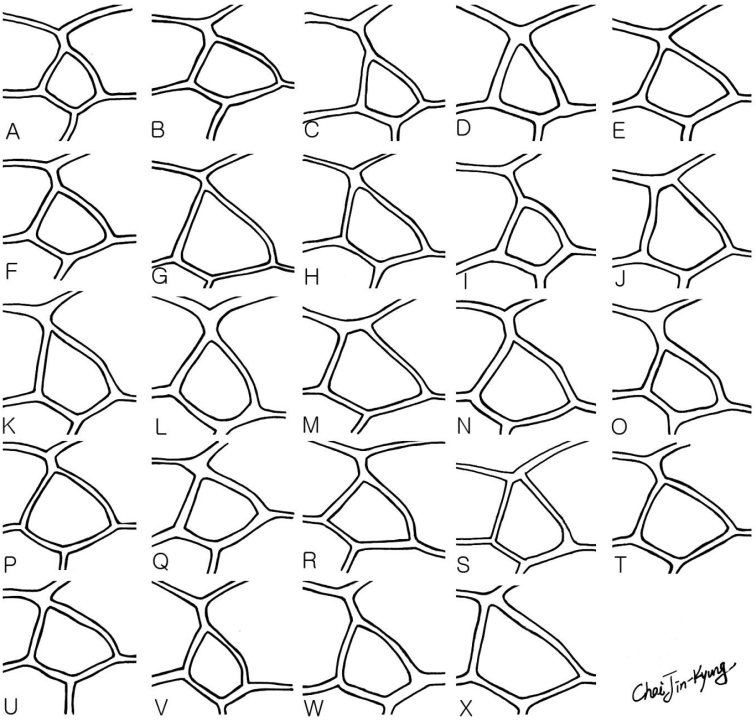
Areolet of fore wing. **A** *Dusona annexa* (=*Dusona americana*) **B** *Dusona celator* **C** *Dusona glauca* **D** *Dusona maruyamator* **E** *Dusona petiolator* **F** *Dusona rugosa* **G** *Dusona falcator* **H** *Dusona matsumurae* **I** *Dusona schikotani* **J** *Dusona signator* **K** *Dusona stragifex* **L** *Dusona ucrainica* **M** *Dusona bellipes* **N** *Dusona bicoloripes* **O** *Dusona chabarowski* **P** *Dusona cultrator* **Q** *Dusona japonica* **R** *Dusona mactatoides* **S** *Dusona scalprata* **T** *Dusona sasayamae* **U** *Dusona obliterata* **V** *Dusona obtutor* **W** *Dusona auriculator* **X** *Dusona longicauda*.

**Figure 6.**
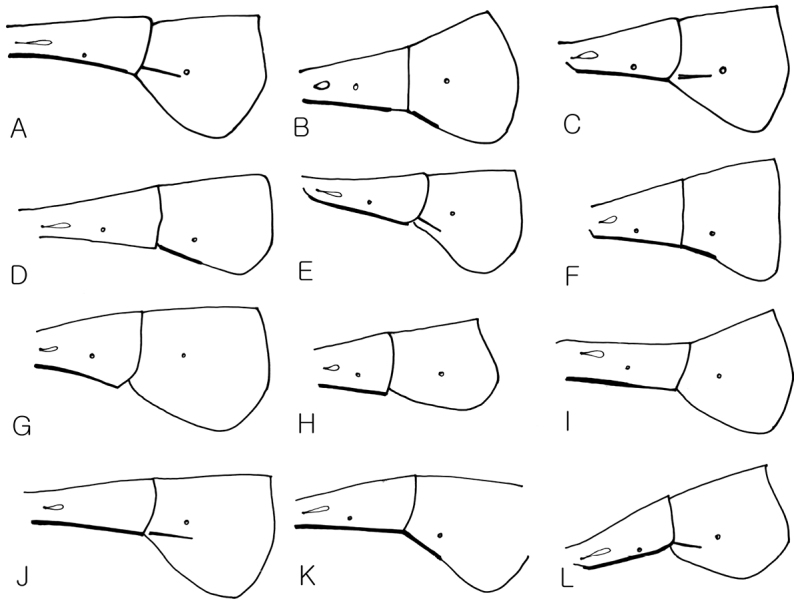
2^nd^ and 3^rd^ terga in lateral view. **A** *Dusona annexa* (=*Dusona americana*) **B** *Dusona celator* **C** *Dusona glauca* **D** *Dusona maruyamator* **E** *Dusona petiolator* **F** *Dusona rugosa* **G** *Dusona falcator* **H** *Dusona matsumurae* **I** *Dusona schikotani* **J** *Dusona signator* **K** *Dusona stragifex* **L** *Dusona ucrainica*.

**Figure 7.**
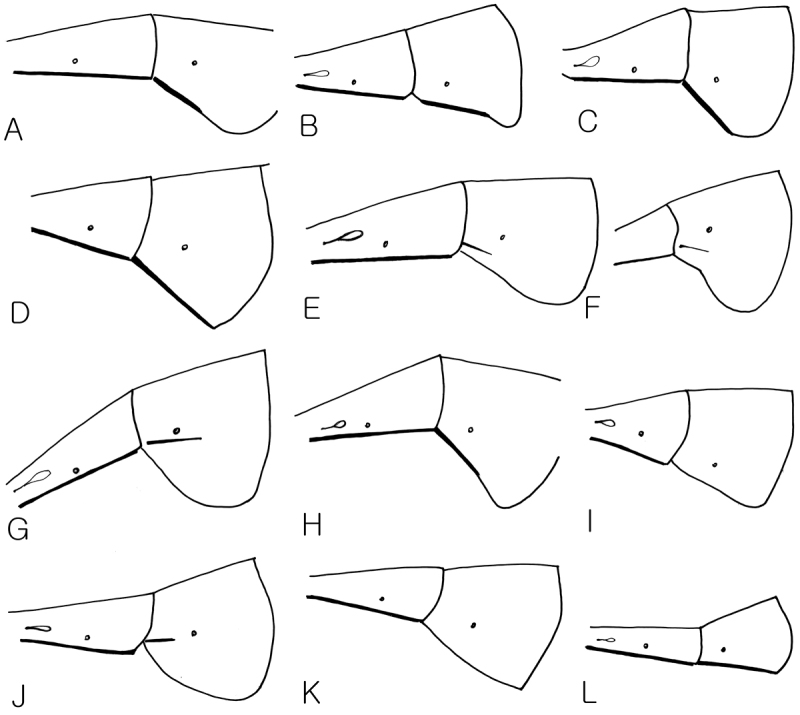
2^nd^ and 3^rd^ terga in lateral view. **A** *Dusona bellipes* **B** *Dusona bicoloripes* **C** *Dusona chabarowski* **D** *Dusona cultrator* **E** *Dusona japonica* **F** *Dusona mactatoides* **G** *Dusona scalprata* **H** *Dusona sasayamae* **I** *Dusona obliterata* **J** *Dusona obtutor* **K** *Dusona auriculator* **L** *Dusona longicauda*.

**Figure 8.**
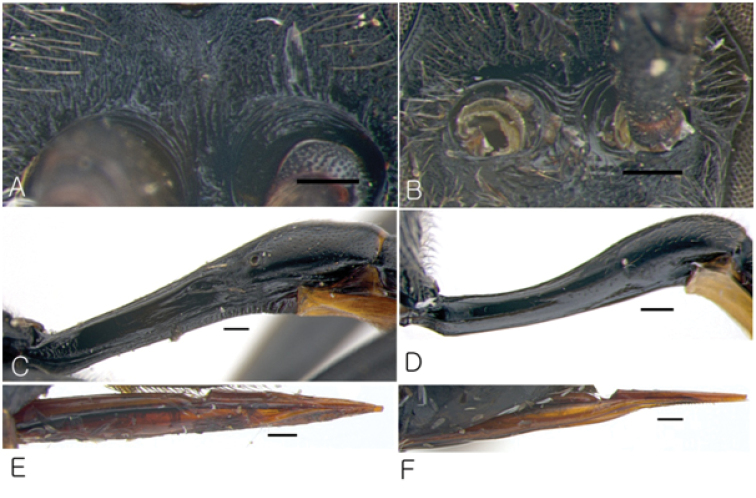
Characters of Korean *Dusona*. **A** Antennal carina highly raised, rim bent upwards and with striae (*Dusona mactatoides*) **B** Antennal carina low and narrow, without striae (*Dusona chabarowski*) **C** Petiole with fine sculpture in front of strong glymma (*Dusona bicoloripes*) **D** Petiole without glymma (*Dusona auriculator*) **E** Lower valve of ovipositor straight (*Dusona koreana*) **F** Lower valve of ovipositor sinuous (*Dusona cultrator*). (Scale bar 0.2 mm).

#### Dusona koreana

Taxon classificationAnimaliaHymenopteraIchneumonidae

Choi & Lee sp. n.

http://zoobank.org/D4E50E9B-BB60-4C79-ABD4-7C3A85BE953D

Fig. 1

##### Holotype.

**Female.** Body length 16.5 mm. Fore wing 13.0 mm.

***Color*:** Face and mesosoma black (Fig. 1B, E). Mandible yellow except black basally (Fig. 1B). Tegula blackish brown. All coxae black except fore coxa yellowish brown apically; fore trochanter to tarsus yellowish brown; mid trochanter and trochantellus black, mid femur to tarsus yellowish brown; hind trochanter to femur black, hind tibia yellowish brown marked dark brown apically. 2^nd^ tergum on apical 0.25, 3^rd^ tergum reddish brown broadly. Ovipositor reddish brown, ovipositor sheath black with brown apically.

***Head*:** Frons slightly depressed, with median longitudinal carina, with transverse wrinkles (Fig. 1C). Antennal flagellum with 61 segments. Antennal carina low and narrow, above with radial wrinkles. Face convex, densely punctate. Clypeus separated from face by weak groove, with truncated apical margin. Mandible tapered to apex, lower tooth as long as upper tooth. Malar space 0.5 times as long as width of mandible. Occipital carina complete. Temple weakly convex.

***Mesosoma*:** Pronotum with distinct epomia. Mesoscutum without notaulus. Speculum depressed and flat, mesopleuron in front of speculum with short longitudinal striae (Fig. 1E). Scutellum and postscutellum convex, scutellum without lateral carina. Propodeal spiracle elongate, distinctly connected to pleural carina. Propodeum depressed, with rugose wrinkles; anterior transverse carina of propodeum present medially, costula absent (Fig. 1D). First radius of fore wing originating before middle of pterostigma. Areolet large, with short stalk (Fig. 1G), 2^nd^ recurrent vein originating before middle of areolet. Nervellus reclivous, intercepted in lower 0.25, discoidella faint (Fig. 1G). Hind wing with 9 distal hamuli. Tibiae and tarsi with short strong spines; tarsal claws pectinate.

***Metasoma*:** Petiole with very strong glymma, dorsolateral carina very strong (Fig. 1F). Epipleurum separated from 3^rd^ tergum, crease with distinct black line. 1^st^ tergum 4.3 times as long as wide, 2^nd^ tergum 1.6 times as long as wide. Ovipositor straight, shorter than hind tibia and longer than hind basitarsus. Ovipositor sheath wide and aciculate basally in lateral view (Fig. 1H).

**Male.** unknown.

##### Intraspecific variation based on the paratypes.

(only differences from holotype described) Antennal flagellum with 56–62 segments.

##### Material examined.

**Holotype: [Korea]** TD: YNU. 1 female, GW, Wonju-si, Panbu-myeon, Seogok-ri, Yongsugol, 19 May 2001, J.W. Lee & D.C. Kim. **Paratype: [Korea]** TD: YNU. 1 female, Seoul, Gangnam-gu, Wonji-dong, 1 June 1985, K.H. Shin; 1 female, GG, Cheonmasan, 7 June 1986, P.C. Yun; 1 female, GG, Seongnam-si, Namhansan, 23 May 1999, E.J. Kim. **Non-Type: [Korea]** TD: YNU. 1 female, 24 May 1986, S.Y. Park.

##### Distribution.

Korea.

##### Region.

Eastern Palaearctic.

##### Host.

Unknown.

##### Etymology.

The specific name is derived from Korea, the country of the type specimens.

##### Remarks.

The species is similar to *Dusona cultrator* (Gravenhorst, 1829) but differs by in the following characters: lower valve of ovipositor straight (Fig. 8E) (lower valve sinuous in *Dusona cultrator*) (Fig. 8F), hind tibia yellowish brown marked dark brown apically (hind tibia yellowish brown, sometimes narrowly marked with brown basally in *Dusona cultrator*); petiole with very large glymma (Fig. 1F), which is bordered by very distinct longitudinal carina dorsally; 1^st^ tergum longer than 4 times as long as wide (1^st^ tergum 3.4 times as long as wide in *Dusona cultrator*). Also, the metasomal segments of *Dusona* are usually reddish brown and partly black but the metasoma of *Dusona koreana* is mostly black with only the 2^nd^ tergum reddish brown apically and 3^rd^ tergum widely reddish brown laterally (Fig. 1A).

#### Dusona bellipes

Taxon classificationAnimaliaHymenopteraIchneumonidae

(Holmgren, 1872)

Figs 3A , 4M , 5M , 7A

Campoplex bellipes Holmgren, 1872: 1–89. Type: male; TD: unknown.
Dusona vernalis Hinz, 1957: 86–90. Holotype: female; TD: ZSM.

##### Material examined.

[Korea]: 1 female, Seoul, Cheonggyesan, 28 May 1989, G.G. Lee.

##### Redescription based on Korean specimen.

**Female.** Body length 16.0 mm.

***Color*:** Face and mesosoma black. Mandible yellow except basal part black. Tegula blackish brown. All coxae black; fore and mid legs yellowish brown except trochanter to femur dark brown ventrally; hind femur black, hind tibia to tarsus dark brown. 2^nd^ tergum on 0.25 apically to 5^th^ tergum reddish brown broadly. Ovipositor reddish brown and ovipositor sheath black except brown apically.

***Head*:** Frons slightly depressed, with fine punctures and with a median longitudinal carina. Antennal flagellum with 57 segments. Antennal carina low and narrow; frons above antennal carina polished. Face moderately convex, densely punctated (Fig. 4M). Clypeus not separated from face, with truncate apical edge. Mandible tapered to apex, lower tooth as long as upper tooth. Malar space much shorter than half of basal width of mandible.

***Mesosoma*:** First radius of fore wing originated before middle of pterostigma. Areolet large (Fig. 5M), 2^nd^ recurrent vein connected before middle of areolet. Nervellus inclivous, intercepted in lower 0.3, discoidella faint. Hind wing with 8 distal hamuli. Propodeum without areola but basal transverse carina distinct, costula absent.

***Metasoma*:** Epipleurum separated from the 3^rd^ tergum, the crease marked with black line (Fig. 7A). 1^st^ tergum 5.1 times as long as wide, 2^nd^ tergum 2.4 times as long as wide. Ovipositor straight and shorter than hind tibia.

##### Distribution.

Korea (new record), Austria, Belarus, France, Germany, Italy, Japan, Latvia, Netherlands, Poland, Romania, Russia (Saratov), Sweden, Switzerland and United Kingdom.

##### Region.

Eastern Palaearctic, Western Palaearctic.

##### Host.

Unknown.

#### Dusona bicoloripes

Taxon classificationAnimaliaHymenopteraIchneumonidae

(Ashmead, 1906)

Figs 3B , 4N , 5N , 7B

Campoplex bicoloripes Ashmead, 1906: 169-201. Type: male. TD: USNM.
Campoplex foersteri Roman 1942: 1-20. Lectotype: female; TD: ZSM.

##### Material examined.

[Korea]: 1 female, Seoul, Seocho-gu, Cheonggyesan, 21 May 2002, H.J. Lim.

##### Redescription based on Korean specimen.

**Female.** Body length 15.5 mm.

***Color*:** Face and mesosoma black. Mandible yellow except basal part black. Tegula black. All coxae black; fore and mid legs brown to dark brown; hind femur black, tibia to tarsus dark brown. 2^nd^ tergum on 0.25 apically to 5^th^ tergum reddish brown, except upper part of half of 5^th^ tergum broadly black. Ovipositor reddish brown and ovipositor sheath black except brown apically.

***Head*:** Frons slightly depressed, with fine punctures and with weak median longitudinal carina. Antennal flagellum with 55 segments. Antennal carina low and narrow, without wrinkles. Face moderately convex, densely punctated (Fig. 4N). Clypeus not separated from face, with truncate apical edge. Mandible tapered to apex, lower tooth as long as upper tooth. Malar space shorter than half of basal width of mandible.

***Mesosoma*:** First radius of fore wing originated from middle of pterostigma. Areolet with long stalk, 2^nd^ recurrent vein connected before middle of areolet (Fig. 5N). Nervellus vertical or reclivous, intercepted in lower 0.3, discoidella faint. Hind wing with 8 distal hamuli. Propodeum without areola but basal transverse carina distinct, costula absent.

***Metasoma*:** Epipleurum separated from the 3^rd^ tergum, the crease marked with black line (Fig. 7B). 1^st^ tergum 3.6 times as long as wide, 2^nd^ tergum 1.8 times as long as wide. Ovipositor straight and shorter than hind tibia.

##### Distribution.

Korea (new record), Algeria, Armenia, Austria, Azerbaijan, Belarus, Belgium, Bulgaria, Croatia, Czech Republic, late Czechoslovakia, Finland, France, Georgia, Germany, Greece, Ireland, Italy, Japan, Kazakhstan, Kyrgyzstan, Moldova, Netherlands, Norway, Poland, Romania, Russia (Kabarovsk, Krasnodar, Primor’ye, Sakhalin, Sankt Petersburg, Yevreyskaya), Slovakia, Slovenia, Spain, Switzerland, Turkey, Ukraine and United Kingdom.

##### Region.

Eastern Palaearctic, Western Palaearctic.

##### Host.

Unknown.

#### Dusona chabarowski

Taxon classificationAnimaliaHymenopteraIchneumonidae

Hinz & Horstmann, 2004

Figs 3C , 4O , 5O , 7C

Dusona chabarowski Hinz & Horstmann, 2004: 59. Holotype: female; TD: ZI.

##### Material examined.

[Korea]: 1 female, Seoul, Nowon-gu, Suraksan, 15 May 1997, J.Y. Kim; 1 female, Seoul, Hwagyesan, 6 May 1989, J.H. Hwang; 1 female, GG Anyang-si, Anyang Arboretum, 13 May 1995, T.H. Gu; 1 female, GW Chuncheon-si, Bongraesan, 13 June 1981, S.H. Kim; 1 female, GW Sockcho-si, Seolaksan National Park, 18 May 2002, M.H. Kim.

##### Redescription based on Korean specimens.

**Female.** Body length 10.0-13.0 mm.

***Color*:** Face and mesosoma black. Mandible dark brown except ventrobasal part black. Tegula blackish brown. All coxae black; fore leg dark reddish brown; mid leg dark brown except tibia brown; hind leg black. 2^nd^ tergum on 0.3 apically to 4^th^ tergum reddish brown. Ovipositor reddish brown and ovipositor sheath black and brown apically.

***Head*:** Frons slightly depressed, with median longitudinal carina. Antennal flagellum with 47-53 segments. Antennal carina low and narrow. Face moderately convex, densely punctated. Clypeus not separated from face, with truncate apical edge (Fig. 4O). Mandible tapered to apex, lower tooth as long as upper tooth. Malar space as long as half of basal width of mandible.

***Mesosoma*:** First radius of fore wing originated before middle of pterostigma. Areolet with long stalk (Fig. 5O), 2^nd^ recurrent vein begin before middle of areolet. Nervellus almost vertical or inclivous, intercepted in lower 0.5, discoidella absent. Hind wing with 8 distal hamuli. Propodeum without areola, costula absent.

***Metasoma*:** Epipleurum separated from the 3^rd^ tergum, the crease with black line (Fig. 7C). 1^st^ tergum 3.1 times as long as wide, 2^nd^ tergum 1.7 times as long as wide. Ovipositor straight and shorter than hind basitarsus.

##### Distribution.

Korea (new record) and Russia (Khabarovsk, Primo’ye).

##### Region.

Eastern Palaearctic.

##### Host.

Unknown.

#### Dusona cultrator

Taxon classificationAnimaliaHymenopteraIchneumonidae

(Gravenhorst, 1829)

Figs 3D , 4P , 5P , 7D

Campoplex cultrator Gravenhorst, 1829: 1-1097. Type: female; TD: MRSN.
Campoplex nigriventris Ulbricht, 1916: 1-21. Type: male; TD: NM.
Campoplex phalerae Uchida, 1929: 169-187. Lectotype: female; TD: HU.

##### Material examined.

[Korea]: 1 female, Seoul, Dobong-gu, Bukhansan National Park, 24 April 1999, T.H. Kim.

##### Redescription based on Korean specimen.

**Female.** Body length 17.5 mm.

***Color*:** Face and mesosoma black. Mandible yellow. Tegula blackish brown. All coxae black; fore leg yellowish brown; mid trochanter and trochantellus black, mid femur brown; hind leg black except tibia brown. 2^nd^ tergum on 0.3 apically to 3^rd^ tergum reddish brown completely. Ovipositor reddish brown.

***Head*:** Frons slightly depressed, with median longitudinal carina. Antennal flagellum all missing. Antennal carina low and narrow; antennal carina above with radial wrinkles. Face moderately convex, densely punctated. Clypeus not separated from face, with truncate apical margin (Fig. 4P). Mandible tapered to apex, lower tooth as long as upper tooth. Malar space shorter than half of basal width of mandible.

***Mesosoma*:** First radius of fore wing originated before middle of pterostigma. Areolet large (Fig. 5P), with short stalk, 2^nd^ recurrent vein begin before middle of areolet. Nervellus vertical, intercepted in lower 0.3, discoidella faint. Hind wing with 10 distal hamuli. Propodeum without areola, costula incomplete.

***Metasoma*:** Epipleurum separated from the 3^rd^ tergum, the crease with black line (Fig. 7D). 1^st^ tergum 3.4 times as long as wide, 2^nd^ tergum 1.2 times as long as wide. Lower valve of ovipositor winding, ovipositor shorter than hind tibia.

##### Distribution.

Korea (new record), Algeria, Austria, Belarus, Belgium, Bulgaria, Czech Republic, late Czechoslovakia, Finland, France, Germany, Greece, Hungary, Italy, Japan, Kazakhstan, Kyrgyzstan, Latvia, Moldova, Morocco, Netherlands, Poland, Romania, Russia (Chita, Irkutsk, Khabarovsk, Primor’ye, Samarskaya, Sankt Petersburg, Tomsk, Yaroslavl), Slovakia, Spain, Sweden, Switzerland, Tunisia, Turkey, Ukraine and United Kingdom.

##### Region.

Eastern Palaearctic, Western Palaearctic.

##### Host.

Lepidoptera. Noctuidae: *Orthosia stabilis* (Denis & Schiffermüller, 1775).

#### Dusona japonica

Taxon classificationAnimaliaHymenopteraIchneumonidae

(Cameron, 1906)

Figs 3E , 4Q , 5Q , 7E

Campoplex japonica Cameron, 1906: 98-99. Holotype: female; TD: NHM.
Dusona interima Gupta & Gupta, 1977: 1-226. Type: female; TD: GUPTA.

##### Material examined.

[Korea]: 1 female, Seoul, Nowon-gu, Hagye1-dong, 25 April 1998, H.J. Yun; 2 females, GB Gyeongsan-si, Dae-dong, Yeungnam univ., 22-29 April 2008, J.W. Lee; 1 female, JN Yeongkwang-gun, Yeomsan-myeon, Bongnam-ri, 23 April 2009, J.K. Choi & D.H. Lee.

##### Redescription based on Korean specimens.

**Female.** Body length 11.0-12.0 mm.

***Color*:** Face and mesosoma black. Mandible dark brown to black. Tegula black. All legs black; fore femur and tibia with yellow spots on anterior part. 3^rd^ tergum and 4^th^ tergum reddish brown, basal part of 5^th^ tergum reddish brown. Ovipositor reddish brown to dark brown and ovipositor sheath black.

***Head*:** Frons not depressed, without median longitudinal carina. Antennal flagellum with 48-52 segments. Antennal carina low and narrow. Face moderately convex, densely punctated. Clypeus a little separated from face by weak groove, with truncate apical margin (Fig. 4Q). Mandible tapered to apex, lower tooth as long as upper tooth. Malar space longer than half of basal width of mandible.

***Mesosoma*:** First radius of fore wing originated from middle of pterostigma. Areolet with short stalk, 2^nd^ recurrent vein begin before middle of areolet (Fig. 5Q). Nervellus inclivous, intercepted in lower 0.1, discoidella absent. Hind wing with 6 distal hamuli. Propodeum without areola, costula absent.

***Metasoma*:** Epipleurum not separated from the 3^rd^ tergum, with lateral black line above the ventrolateral edge (Fig. 7E). 1^st^ tergum 4.6 times as long as wide, 2^nd^ tergum 1.9 times as long as wide. Ovipositor shorter than hind basitarsus.

##### Distribution.

Korea (new record), China, India, Japan, Kazakhstan, Kyrgyzstan, Russia (Irkutsk, Sakhalin) and Uzbekistan.

##### Region.

Eastern Palaearctic, Oriental.

##### Host.

Unknown.

#### Dusona mactatoides

Taxon classificationAnimaliaHymenopteraIchneumonidae

Hinz, 1994

Figs 3F , 4R , 5R , 7F

Dusona mactatoides Hinz, 1994: 29-46. Type: female; TD: ZI.

##### Material examined.

[Korea]: 1 female, GG Hanam-si, Namhansanseong, 31 July 1993, J.S. Lee.

##### Redescription based on Korean specimen.

**Female.** Body length 17.5 mm.

***Color*:** Face and mesosoma black. Mandible brown except ventrobasal part dark brown. Tegula blackish brown. All coxae black; fore leg yellowish brown; mid femur borwn to blackish brown, mid tibia yellowish brown, mid tarsi dark brown; hind leg black except tibia brown and black apically. 2^nd^ tergum on ventro-apical part reddish brown; 3^rd^ tergum to 7^th^ tergum dark reddish brown. Ovipositor sheath black.

***Head*:** Frons and surface between the antennal sockets deeply depressed, with median longitudinal carina. Antennal flagellum with 64 segments. Antennal carina strongly raised, the rim bend upwards and with transverse striae; antennal carina above with long radial wrinkles. Face moderately convex, densely punctated. Central part of face with weak protuberance (Fig. 4R). Clypeus weakly convex and a little separated from face by a weak groove, with truncate apical margin. Mandible tapered to apex, lower tooth as long as upper tooth. Malar space shorter than half basal width of mandible.

***Mesosoma*:** First radius of fore wing originated from middle of pterostigma. Areolet with stalk, 2^nd^ recurrent vein begin before middle of areolet (Fig. 5R). Nervellus reclivous, intercepted in lower 0.3, discoidella faint. Hind wing with 8 distal hamuli. Propodeum without areola, costula incomplete.

***Metasoma*:** Epipleurum separated from the 3^rd^ tergum, but the crease without black line, but with lateral black line above the anterior ventrolateral edge (Fig. 7F). 1^st^ tergum 4.3 times as long as wide, 2^nd^ tergum 2.2 times as long as wide. Ovipositor shorter than hind basitarsus.

##### Distribution.

Korea (new record) and Russia (Sakhalin).

##### Region.

Eastern Palaearctic.

##### Host.

Unknown.

#### Dusona scalprata

Taxon classificationAnimaliaHymenopteraIchneumonidae

Horstmann, 2004

Figs 3G , 4S , 5S , 7G

Dusona scalprata Horstmann, 2004: 149. Holotype: male; TD: MLSU.

##### Material examined.

[Korea]: 1 male, Seoul, Gangnam-gu, Suseo-dong, Guryongsan, 27 May 1998, J.E. Kim.

##### Redescription based on Korean specimen.

**Male.** Body length 15.5 mm.

***Color*:** Face and mesosoma black. Mandible yellow except basal part black. Tegula blackish brown. Fore and mid legs yellowish brown except coxae black; hind coxa to femur black except apical part of femur yellowish brown, hind tibia and tarsus yellowish brown. 2^nd^ tergum on ventro-apical to 7^th^ tergum reddish brown; 2^nd^ tergum broadly black dorsally, 3^rd^ to 7^th^ terga with narrow black line dorsally. Clasper of male reddish brown.

***Head*:** Frons slightly depressed, with fine punctures and with median longitudinal carina. Antennal flagellum with 53+ segments, apical flagellomeres missing. Antennal carina low and narrow. Face moderately convex, densely punctated, with white hairs (Fig. 4S). Clypeus a little separated from face by weak groove, with truncate apical margin. Mandible tapered to apex, lower tooth a little shorter than upper tooth. Malar space as long as half of basal width of mandible.

***Mesosoma*:** First radius of fore wing originated from middle of pterostigma. Areolet without stalk, 2^nd^ recurrent vein begin before middle of areolet (Fig. 5S). Nervellus inclivous, intercepted in lower 0.25, discoidella absent. Hind wing with 8 distal hamuli. Propodeum without areola but basal transverse carina distinct.

***Metasoma*:** Epipleurum not separated from the 3^rd^ tergum, with indistinct lateral black line above the anterior ventrolateral edge (Fig. 7G). 1^st^ tergum 4.4 times as long as wide, 2^nd^ tergum 4.6 times as long as wide.

##### Distribution.

Korea (new record) and Russia (Primor’ye).

##### Region.

Eastern Palaearctic.

##### Host.

Unknown.

#### Dusona sasayamae

Taxon classificationAnimaliaHymenopteraIchneumonidae

Hinz & Horstmann, 2004

Figs 3H , 4T , 5T , 7H

Dusona sasayamae Hinz & Horstmann, 2004: 148. Holotype: female; TD: HU.

##### Material examined.

[Korea]: 2 females, Daejeon-si, Dong-gu, Daejeon univ., 16 May–5 June 2006, J.W. Lee.

##### Redescription based on Korean specimens.

**Female.** Body length 12.0–16.0 mm.

***Color*:** Face and mesosoma black. Mandible yellow. Tegula blackish brown. All coxae black; fore leg yellowish brown except outer areas of trochanter and inner areas of trochantellus blackish brown; mid leg blackish brown except part of femur and tibia brown; hind leg black except tibia brown. 2^nd^ tergum to 4^th^ tergum reddish brown, 5^th^ tergum reddish brown except black dorsally, 6^th^ tergum black except reddish brown ventrally. Ovipositor reddish brown and ovipositor sheath black and reddish brown apically.

***Head*:** Frons slightly depressed, with fine punctures and with median longitudinal carina. Antennal flagellum with 55 segments. Antennal carina low and narrow. Face moderately convex, densely punctated. Clypeus not separated from face, with concaved apical edge (Fig. 4T). Mandible tapered to apex, lower tooth shorter than upper tooth. Malar space shorter than half basal width of mandible.

***Mesosoma*:** First radius of fore wing originated from middle of pterostigma. Areolet with stalk (Fig. 5T), 2^nd^ recurrent vein begin a little before middle of areolet. Nervellus almost vertical, intercepted in lower 0.25, discoidella faint. Hind wing with 8 distal hamuli. Propodeum without areola, costula absent.

***Metasoma*:** Epipleurum separated from the 3^rd^ tergum, the crease marked with black line (Fig. 7H). 1^st^ tergum 3.5 times as long as wide, 2^nd^ tergum 1.8 times as long as wide. Ovipositor straight and shorter than hind basitarsus.

##### Distribution.

Korea (new record) and Japan.

##### Region.

Eastern Palaearctic.

##### Host.

Unknown.

#### Dusona obliterata

Taxon classificationAnimaliaHymenopteraIchneumonidae

(Holmgren, 1872)

Figs 3I , 4U , 5U , 7I

Campoplex oblitera Holmgren, 1872: 1-89. Lectotype: female; TD: NR.
Campoplex limiventris Kriechbaumer, 1883: 97-115. Holotype: male; TD: ZSM.

##### Material examined.

[Korea]: 1 female, GG Paju-si, Jeokseong-myeon, Seolma-ri, 18 August 1984, M.I. Lee.

##### Redescription based on Korean specimen.

**Female.** Body length 16.0 mm.

***Color*:** Face and mesosoma black. Mandible yellow. Tegula blackish brown. All coxae black; fore and mid legs yellowish brown; hind leg blackish brown except tibia yellowish brown. 2^nd^ tergum on ventro-apical edge to basal of 5^th^ tergum reddish brown.

***Head*:** Frons slightly depressed, with fine punctures and with partly obliterated median longitudinal carina. Antennal flagellum with 58 segments. Antennal carina low, the rim weak bent upwards and with radial wrinkles. Face moderately convex, densely punctated. Central part of face with weak protuberance (Fig. 4U). Clypeus not separated from face, with truncate apical edge. Mandible tapered to apex, lower tooth as long as upper tooth. Malar space shorter than half basal width of mandible.

***Mesosoma*:** First radius of fore wing originated from middle of pterostigma. Areolet large without stalk, 2^nd^ recurrent vein begin a little before middle of areolet (Fig. 5U). Nervellus vertical, intercepted in lower 0.3, discoidella faint. Hind wing with 9 distal hamuli. Propodeum without areola but basal transverse carina distinct.

***Metasoma*:** Epipleurum not separated from the 3^rd^ tergum, without black line (Fig. 7I). 1^st^ tergum 4.6 times as long as wide, 2^nd^ tergum 2.0 times as long as wide. Ovipositor straight and shorter than hind basitarsus.

##### Distribution.

Korea (new record), Austria, Belarus, Belgium, Bulgaria, Croatia, Czech Republic, late Czechoslovakia, Finland, France, Germany, Hungary, Italy, Latvia, Moldova, Netherlands, Poland, Romania, Russia (Irkutsk, Moscow, Primor’ye, Sankt Petersburg, Smolensk), Spain, Sweden, Switzerland, Turkey and United Kingdom.

##### Region.

Eastern Palaearctic, Western Palaearctic.

##### Host.

Lepidoptera. Noctuidae: *Acronicta leporina* (Linnaeus, 1758); Notodontidae: *Euchila palpina* (Clerck, 1759); Thyatridae: *Achlya flavicornis* (Linnaeus, 1758).

#### Dusona obtutor

Taxon classificationAnimaliaHymenopteraIchneumonidae

Hinz, 1994

Figs 3J , 4V , 5V , 7J

Dusona obtutor Hinz, 1994: 29-46. Holotype: female; TD: HU.

##### Material examined.

[Korea]: 1 female, JB Jeongeup-si, Naejang-dong, Naejangsan National Park, Ansambatsil, 18 May 2004, J.G. Han.

##### Redescription based on Korean specimen.

**Female.** Body length 11.0 mm.

***Color*:** Face and mesosoma black. Mandible yellow except basal part black. Tegula blackish brown. All coxae to trochentellus black; fore femur yellowish brown to dark brown, from fore tibia yellow; middle leg blackish brown except tibia brown; hind leg black. 2^nd^ tergum on ventroapical edge to 3^rd^ tergum reddish brown. Ovipositor reddish brown and ovipositor sheath black.

***Head*:** Frons slightly depressed, with fine punctures and with median longitudinal carina. Antennal flagellum with 43 segments. Antennal carina low and narrow. Face moderately convex, densely punctated (Fig. 4V). Clypeus not separated from face, with truncate apical edge. Mandible tapered to apex, lower tooth shorter than upper tooth. Malar space longer than half basal width of mandible.

***Mesosoma*:** First radius of fore wing originated from middle of pterostigma. Areolet small with long stalk (Fig. 5V), 2^nd^ recurrent vein begin after middle of areolet. Nervellus reclivous, intercepted in lower 0.25, discoidella absent. Hind wing with 7 distal hamuli. Propodeum without areola, basal transverse carina incomplete.

***Metasoma*:** Epipleurum not separated from the 3^rd^ tergum, with distinct lateral black line above the anterior ventrolateral edge (Fig. 7J). 1^st^ tergum 4.4 times as long as wide, 2^nd^ tergum 2.1 times as long as wide. Ovipositor straight, shorter than hind basitarsus.

##### Distribution.

Korea (new record) and Japan.

##### Region.

Eastern Palaearctic.

##### Host.

Unknown.

#### Dusona auriculator

Taxon classificationAnimaliaHymenopteraIchneumonidae

Aubert, 1964

Figs 3K , 4W , 5W , 7K

Dusona auriculator Aubert, 1964: 35–40. Holotype: female; TD: MZ.

##### Material examined.

[Korea]: 1 male, GW Wongju-si, Socho-myeon, Hakgong-ri, Chiaksan National Park, 28 August-16 September 2013, J.W. Lee.

##### Redescription based on Korean specimen.

**Male.** Body length 8.5 mm.

***Color*:** Face and mesosoma black. Mandible and tegula yellow. Fore leg yellow except fore coxa black basally; middle leg yellow except coxa black dorsally; hind coxa black; hind trochanter to tibia brown, femur and tibia black apically; hind tarsus darker than hind femur. 2^nd^ tergum reddish brown on ventroapical edge; 3^rd^ tergum to 7^th^ tergum reddish brown, with narrow black line dorsally. Clasper of male yellowish brown.

***Head*:** Frons slightly depressed, with fine punctures and with median longitudinal carina. Antennal flagellum with 46 segments. Antennal carina strongly raised, the rim widened to a smooth bulge. Face moderately convex, densely punctated. Clypeus not separated from face, with round apical edge (Fig. 4W). Mandible tapered to apex, lower tooth as long as upper tooth. Malar space as long as half basal width of mandible.

***Mesosoma*:** Pronotum and speculum of mesosoma polished. First radius of fore wing originated weakly before middle of pterostigma. Areolet small with long stalk, 2^nd^ recurrent vein distad of middle of areolet (Fig. 5W). Nervellus reclivous, not intercepted or weak, discoidella absent. Hind wing with 5 distal hamuli. Propodeum without areola but basal transverse carina distinct.

***Metasoma*:** Epipleurum not separated from the 3^rd^ tergum, without lateral black line (Fig. 7K). 1^st^ tergum 4.8 times as long as wide, 2^nd^ tergum 2.9 times as long as wide.

##### Distribution.

Korea (new record), Austria, Bulgaria, France, Greece, Italy, Japan, Romania and Russia (Khabarovsk, Primor’ye).

##### Region.

Eastern Palaearctic, Western Palaearctic.

##### Host.

Unknown.

#### Dusona longicauda

Taxon classificationAnimaliaHymenopteraIchneumonidae

(Uchida, 1928)

Figs 3L , 4X , 5X , 7L

Campoplex longicauda Uchida, 1928: 177–297. Holotype: female; TD: HU.

##### Material examined.

[Korea]: 1 female, Seoul, Gangnam-gu, Cheonggyesan, 6 September 1986, Y.H. Lee; 2 females, GG Su-dong, Chukryeongsan, 28 September 1980, H.K. Park; 1 female, GW Sokcho-si, Seorak-dong, 11 June 1992, J.W. Lee; 1 female, GB Gyeongsan-si, Dae-dong, Yeungnam univ., 17 May 1989, Y.K. Lee; 1 female, ditto, 30 May 1989, J.W. Lee; 1 female, ditto, 21-27 May 2008, J.W. Lee; 9 females, GB Eulseong-gun Ansa-myeon, Ansamyeonsamuso, 1 April-1 May 2013, S.J. Park; 1 female, GB Cheongdo-gun, Gakbuk-myeon, Namsan 3-ri, 2-15 June 2008, J.W. Lee; 2 females, GN Jinju-si, Gajwa-dong, 18-24 May 1990, J.W. Lee; 1 female, ditto, 27 May 1991, J.W. Lee; 1 female, ditto, 29 May 1991, J.W. Lee; 1 female, ditto, 1-9 June 1990, J.W. Lee; 1 female, GN Haman-gun, Daesan-myeon, 22 April 1991, J.W. Lee; 1 female, JN Wando-gun Soan-myeon, Soan-do, 15 May-11 June 2011, J.W. Lee; 1 female, JJ Jeju-si, Aewol-eup, Gwangnyeong-ri, Sumeunmulbaengdwi, weltland of 1,100Goji, 24 August 2010, H.S. Lee; 1 female, JJ Seogeypo-si, Cheongsonyeonyayeongjang, 21 May 2003, J.W. Lee.

##### Redescription based on Korean specimens.

**Female.** Body length 10.0–13.0 mm.

***Color*:** Face and mesosoma black. Mandible yellow except basal part black. Tegula blackish brown. All coxae to trochentellus black; fore leg yellowish brown except femur ventrally dark brown; middle leg blackish brown except tibia yellowish brown; hind leg blackish brown. 2^nd^ tergum on 0.25 apically to 4^th^ tergum reddish brown. Ovipositor reddish brown and ovipositor sheath black.

***Head*:** Frons slightly depressed, with fine punctures and with median longitudinal carina. Antennal flagellum with 41-45 segments. Antennal carina low and narrow. Face moderately convex, densely punctated (Fig. 4X). Clypeus not separated from face, with round apical edge. Mandible tapered to apex, lower tooth as long as upper tooth. Malar space as long as half basal width of mandible.

***Mesosoma*:** First radius of fore wing originated weakly before middle of pterostigma. Areolet large, without stalk (Fig. 5X), 2^nd^ recurrent vein begin a little before middle of areolet. Nervellus inclivous, intercepted in lower 0.5, discoidella faint. Hind wing with 6-7 distal hamuli. Propodeum without areola but basal transverse carina distinct.

***Metasoma*:** Epipleurum separated from the 3^rd^ tergum, the crease marked with black line (Fig. 7L). 1^st^ tergum 5.2 times as long as wide, 2^nd^ tergum 2.4 times as long as wide. Ovipositor upcurved, longer than hind tibia.

##### Distribution.

Korea (new record), China, Japan and Russia (Khabarovsk, Krasnoyarsk, Primor’ye, Sakhalin).

##### Region.

Eastern Palaearctic, Oriental.

##### Host.

Unknown.

#### Dusona annexa

Taxon classificationAnimaliaHymenopteraIchneumonidae

(Förster, 1868)

Figs 2A , 4A , 5A , 6A

Campoplex annexa Förster, 1868: 761–876. Type: female; TD: ZSM.
Casinaria americana Ashmead, 1890: 1–47. Type: female; TD: USNM.
Campoplegidea erythromera Viereck, 1926: 173–186. Type: female; TD: CNC.
Campoplex neoluteipes Uchida, 1942: 107–146. Type: female; TD: HU.
Dusona oyamadai Hinz, 1994: 29–46. Type: female; TD: lost.

##### Material examined.

[Korea]: No specimens; [Germany]: 1 female, 17 July 1956, Zwiesel B.W.

##### Distribution.

Korea, Austria, Belarus, Belgium, Bulgaria, Canada, China, Czech Republic, late Czechoslovakia, Finland, France, Georgia, Germany, Hungary, Ireland, Italy, Japan, Kazakhstan, Moldova, Mongolia, Netherlands, Norway, Poland, Romania, Russia (Amur, Buryatskaya Respublika, Chita, Kamchatka, Khabarovsk, Murmansk, Primor’ye, Sakhalin, Sankt Petersburg, Yevreyskaya), Slovakia, Sweden, Switzerland, Turkey, U.S.A., Ukraine and United Kingdom.

##### Region.

Eastern Palaearctic, Western Palaearctic, Nearctic.

##### Host.

Unknown.

##### Remarks.

No Korean specimens were available for this study. However we have seen a voucher specimen from ZSM.

#### Dusona celator

Taxon classificationAnimaliaHymenopteraIchneumonidae

Hinz, 1985

Figs 2B , 4B , 5B , 6B

Dusona celator Hinz, 1985: 297–317. Type: female; TD: ZI.

##### Material examined.

[Korea]: No specimens; [TD: ZSM]: 1 female.

##### Distribution.

Korea and Russia (Chita, Primor’ye).

##### Region.

Eastern Palaearctic.

##### Host.

Unknown.

##### Remarks.

No Korean specimens were available for this study. However we have seen a Russian voucher specimen from ZSM. The tip of the fore coxa of male is yellowish red, whereas in other characters it is similar to the female.

#### Dusona crassiventris

Taxon classificationAnimaliaHymenopteraIchneumonidae

Horstmann, 2004

Dusona crassiventris Horstmann, 2004: 67. Holotype: female; TD: SAWON.

##### Material examined.

[Korea]: No specimens.

##### Distribution.

Korea.

##### Region.

Eastern Palaearctic.

##### Host.

Unknown.

##### Remarks.

This species was recorded from Korea as an endemic species by Horstmann (2004). However no Korean specimens were available for this study.

#### Dusona falcator

Taxon classificationAnimaliaHymenopteraIchneumonidae

(Fabricius, 1775)

Figs 2G , 4G , 5G , 6G

Ichneumon falcator Fabricius, 1775: 832. Holotype: female; TD: UZM.

##### Material examined.

[Korea]: 1 male, GW Donghae-si, Samhwa-dong, Muryeong valley, 16–28 June 2005, J.W. Lee; 1 female, JN Jeongeup-si Ibam-myeon, Deungcheon-ri, 23 July 2004, J.G. Han.

##### Distribution.

Korea, Austria, Azerbaijan, Belgium, Bulgaria, Czech Republic, late Czechoslovakia, Finland, France, Germany, Hungary, Ireland, Israel, Italy, Kazakhstan, Netherlands, Norway, Poland, Romania, Russia (Altayskiy, Kirov, Primor’ye, Samarskaya, Sankt Petersburg), Sweden, Switzerland, Turkey, Ukraine and United Kingdom.

##### Region.

Eastern Palaearctic, Western Palaearctic.

##### Host.

Unknown.

#### Dusona glauca

Taxon classificationAnimaliaHymenopteraIchneumonidae

(Norton, 1863)

Figs 2C , 4C , 5C , 6C

Campoplex glauca Norton, 1863: 357–368. Type: female; TD: YU.
Campoplex dissitus Norton, 1863: 357–368. Type: female; TD: MCZ.
Campoplegidea rossi Viereck, 1925: 259–273. Type: male; TD: CNC.

##### Material examined.

[Korea]: No specimens; [TD: ZSM]: 1 male.

##### Distribution.

Korea, Canada, Japan, Russia (Chita, Irkutsk, Khabarovsk, Magadanskaya, Primor’ye, Sakhalin) and U.S.A.

##### Region.

Eastern Palaearctic, Nearctic.

##### Host.

Unknown.

##### Remarks.

No Korean specimens were available for this study. However we have seen a Japanese voucher specimen from ZSM.

#### Dusona maruyamator

Taxon classificationAnimaliaHymenopteraIchneumonidae

Hinz, 1979

Figs 2D , 4D , 5D , 6D

Dusona maruyamator Hinz, 1979: 215. Type: female; TD: HU.

##### Material examined.

[Korea]: 4 females, Daejeon-si, Daejeon univ., 1-17 May 2006, J.K Choi; 1 female, GB Gyeongsan-si, Dae-dong, Yeungnam univ., 22 April-1 May 2006, J.W. Lee; 1 female, JB Jeongeup-si, Naejang-dong, Wonjeogam, 28 April-28 May 2006, J.K Choi.

##### Distribution.

Korea, Japan and Russia (Khabarovsk, Primor’ye, Sakhalin).

##### Region.

Eastern Palaearctic.

##### Host.

Unknown.

#### Dusona matsumurae

Taxon classificationAnimaliaHymenopteraIchneumonidae

(Uchida, 1928)

Figs 2H , 4H , 5H , 6H

Campoplex matsumurae Uchida, 1928: 277.
Mesochorus japonicus Matsumura 1912: 117. Lectotype: female; TD: HU.

##### Material examined.

[Korea]: 1 female, GG Gapyeung-gun, Seorak-myeon, 14 June 1992, J.W. Lee; 1 female, 1 male, GN Gayasan, 5 August 1960, C.H. Kim.

##### Distribution.

Korea, Japan and Russia (Amur, Primor’ye).

##### Region.

Eastern Palaearctic.

##### Host.

Unknown.

#### Dusona okadai

Taxon classificationAnimaliaHymenopteraIchneumonidae

(Uchida, 1942)

Campoplex okadai Uchida, 1942: 136. Type: female; TD: HU.

##### Material examined.

[Korea]: No specimens; Holotype: 1 female.

##### Distribution.

Korea, China and Russia (Primor’ye, Yevreyskaya).

##### Region.

Eastern Palaearctic.

##### Host.

Unknown.

##### Remarks.

No Korean specimens were available for this study. However we have seen a voucher specimen from ZSM and have loaned holotype from HU.

#### Dusona petiolator

Taxon classificationAnimaliaHymenopteraIchneumonidae

(Fabricius, 1804)

Figs 2E , 4E , 5E , 6E

Ophion petiolator Fabricius, 1804: 140. Holotype: female; TD: UZM.
Campoplex lapponicus (Holmgren, 1860): 37. Type: female; TD: NR.
Campoplex callizonus Förster, 1868: 761-876. Type: female; TD: ZSM.
Campoplex greeni (Cameron, 1905): 127. Type: female; TD: NHM.
Campoplex sachalinensis Uchida, 1928: 276. Lectotype: female; TD: HU.

##### Material examined.

[Korea]: 1 female, 1male, Daejeon-si, Daejeon univ., 16 May–5 June 2006, J.W. Lee; 1 male, ditto, 1-17 May 2006, J.W. Lee.

##### Distribution.

Korea, Austria, Belarus, Belgium, Canada, Czech Republic, late Czechoslovakia, Denmark, Finland, France, Germany, India, Ireland, Italy, Japan, Kyrgyzstan, Latvia, Netherlands, Norway, Pakistan, Poland, Romania, Russia (Altayskiy, Amur, Chita, Irkutsk, Kamchatka, Khabarovsk, Murmansk, Novosibirsk, Orenburg, Sakhalin, Sankt Petersburg, Vologda, Yaroslavl), Sri Lanka, Sweden, Turkey, U.S.A. and United Kingdom.

##### Region.

Eastern Palaearctic, Western Palaearctic, Nearctic, Oriental.

##### Host.

Lepidoptera. Geometridae: *Philereme transversata* (Hufnagel, 1767), *Philereme vetulata* (Denis & Schiffermüller, 1775), *Rheumaptera cervinalis* (Scopoli, 1763), *Rheumaptera hastata* (Linnaeus, 1758), *Rheumaptera undulata* (Linnaeus, 1758).

#### Dusona rugosa

Taxon classificationAnimaliaHymenopteraIchneumonidae

Horstmann, 2004

Figs 2F , 4F , 5F , 6F

Dusona rugosa Horstmann, 2004: 146. Holotype: female; TD: HU.

##### Material examined.

[Korea]: No specimens; Holotype: 1 female.

##### Distribution.

Korea and Japan.

##### Region.

Eastern Palaearctic.

##### Host.

Unknown.

##### Remarks.

No Korean specimens were available for this study. However we have seen a voucher specimen from ZSM and have loaned holotype from HU.

#### Dusona schikotani

Taxon classificationAnimaliaHymenopteraIchneumonidae

Hinz, 1994

Figs 2I , 4I , 5I , 6I

Dusona schikotani Hinz, 1994: 29–46. Holotype: female; TD: ZI.

##### Material examined.

[Korea]: No specimens.

##### Distribution.

Korea and Russia (Primor’ye, Sakhalin).

##### Region.

Eastern Palaearctic.

##### Host.

Unknown.

##### Remarks.

No Korean specimens were available for this study. However we have seen a voucher specimen in ZSM.

#### Dusona signator

Taxon classificationAnimaliaHymenopteraIchneumonidae

(Brauns, 1895)

Figs 2J , 4J , 5J , 6J

Campoplex signator Brauns, 1895: 42–49. Type: female; TD: TMA.
Campoplex jozanus Uchida 1928: 275. Type: female; TD: HU.
Campoplex subrubrus Uchida 1928: 275. Lectoype: female; TD: HU.
Campoplex ohshimensis Uchida 1930: 78–88. Type: female; TD: HU.
Campoplex kaigensis Uchida 1942: 135. Type: female; TD: HU.

##### Material examined.

[Korea]: 17 males, Daejeon-si, Dong-gu, Daejeon univ., 1–17 May 2006, J.W. Lee; 1 male, GB Gyeongsan-si, Dae-dong, Yeungnam univ., 16 May 1989, J.H. Park..

##### Distribution.

Korea, Austria, Bulgaria, China, Czech Republic, late Czechoslovakia, France, Georgia, Germany, Hungary, Italy, Japan, Moldova, Poland, Romania, Russia (Primor’ye, Sakhalin, Yevreyskaya), Slovakia and Slovenia.

##### Region.

Eastern Palaearctic, Western Palaearctic.

##### Host.

Unknown.

#### Dusona stragifex

Taxon classificationAnimaliaHymenopteraIchneumonidae

(Förster, 1868)

Figs 2K , 4K , 5K , 6K

Campoplex stragifex Förster, 1868: 811. Type: female; TD: ZSM.
Campoplex adjunctus Förster 1868: 761–876. Type: female; TD: ZSM.
Campoplex areolatus Brauns 1895: 42–49. Type: male; TD: TMA.
Campoplex daisetsuzanus Uchida 1928: 277. Lectotype: female; TD: HU.

##### Material examined.

[Korea]: No specimens.

##### Distribution.

Korea, Armenia, Austria, Belarus, Belgium, Bulgaria, Czech Republic, late Czechoslovakia, Finland, France, Georgia, Germany, Greece, Hungary, Ireland, Italy, Japan, Latvia, Moldova, Morocco, Netherlands, Norway, Poland, Romania, Russia (Altayskiy, Astrakhanskaya, Buryatskaya, Khabarovsk, Murmansk, Primor’ye, Sakhalin, Sverdlovsk, Tambov, Tomsk), Slovakia, Spain, Sweden, Switzerland, Turkey, Ukraine and United Kingdom.

##### Region.

Eastern Palaearctic, Western Palaearctic.

##### Host.

Lepidoptera. Geometridae: *Lycia isabellae* (Harrison, 1914), *Odontopera bidentata* (Clerck, 1759); Noctuidae: *Lithophane ornitopus* (Hufnagel, 1766), *Orthosia opima* (Hübner, 1809), *Polymixis flavicincta* (Denis & Schiffermüller, 1775).

##### Remarks.

No Korean specimens were available for this study. However we have seen a voucher specimen in ZSM. This species is very similar to *Dusona bicoloripes* and *Dusona chabrowski*, but the impression of *Dusona stragifex* in front of the speculum is distinctly striate, the propodeum is distinctly depressed, and the longitudinal carinae are present medially and posteriorly.

#### Dusona ucrainica

Taxon classificationAnimaliaHymenopteraIchneumonidae

Hinz, 1972

Figs 2L , 4L , 5L , 6L

Dusona ucrainica Hinz, 1972: 45–54. Type: female; TD: DEI.

##### Material examined.

[Korea]: No specimens.

##### Distribution.

Korea, Armenia, Austria, Azerbaijan, Bulgaria, Croatia, late Czechoslovakia, Japan, Moldova, Mongolia, Romania, Russia (Amur, Chita, Omsk, Primor’ye, Volgograd, Yevreyskaya), Slovakia, Turkey and Ukraine.

##### Region.

Eastern Palaearctic, Western Palaearctic.

##### Host.

Lepidoptera. Geometridae: *Tephrina murinaria* (Denis & Schiffermüller, 1775); Noctuidae: *Discestra trifolii* (Hufnagel, 1766), *Heliothis viriplaca* (Hufnagel, 1766), *Mamestra brassicae* (Linnaeus, 1758).

##### Remarks.

No Korean specimens were available for this study. However we have seen a voucher specimen in ZSM.

## Supplementary Material

XML Treatment for Dusona

XML Treatment for Dusona koreana

XML Treatment for Dusona bellipes

XML Treatment for Dusona bicoloripes

XML Treatment for Dusona chabarowski

XML Treatment for Dusona cultrator

XML Treatment for Dusona japonica

XML Treatment for Dusona mactatoides

XML Treatment for Dusona scalprata

XML Treatment for Dusona sasayamae

XML Treatment for Dusona obliterata

XML Treatment for Dusona obtutor

XML Treatment for Dusona auriculator

XML Treatment for Dusona longicauda

XML Treatment for Dusona annexa

XML Treatment for Dusona celator

XML Treatment for Dusona crassiventris

XML Treatment for Dusona falcator

XML Treatment for Dusona glauca

XML Treatment for Dusona maruyamator

XML Treatment for Dusona matsumurae

XML Treatment for Dusona okadai

XML Treatment for Dusona petiolator

XML Treatment for Dusona rugosa

XML Treatment for Dusona schikotani

XML Treatment for Dusona signator

XML Treatment for Dusona stragifex

XML Treatment for Dusona ucrainica
